# Emotion regulation: exploring the impact of stress and sex

**DOI:** 10.3389/fnbeh.2014.00397

**Published:** 2014-11-13

**Authors:** Valerie L. Kinner, Serkan Het, Oliver T. Wolf

**Affiliations:** Department of Cognitive Psychology, Institute of Cognitive Neuroscience, Ruhr University BochumBochum, Germany

**Keywords:** cortisol, emotion regulation, stress, socially evaluated cold pressor test, sex differences

## Abstract

Emotion regulation is a major prerequisite for adaptive behavior. The capacity to regulate emotions is particularly important during and after the encounter of a stressor. However, the impact of acute stress and its associated neuroendocrine alterations on emotion regulation have received little attention so far. This study aimed to explore how stress-induced cortisol increases affect three different emotion regulation strategies. Seventy two healthy men and women were either exposed to a stressor or a control condition. Subsequently participants viewed positive and negative images and were asked to up- or down-regulate their emotional responses or simultaneously required to solve an arithmetic task (distraction). The factors stress, sex, and strategy were operationalized as between group factors (*n* = 6 per cell). Stress caused an increase in blood pressure and higher subjective stress ratings. An increase in cortisol was observed in male participants only. In contrast to controls, stressed participants were less effective in distracting themselves from the emotional pictures. The results further suggest that in women stress enhances the ability to decrease negative emotions. These findings characterize the impact of stress and sex on emotion regulation and provide initial evidence that these factors may interact.

## Introduction

The ability to regulate emotions is essential for mental and physical health, especially in the face of aversive events. Emotions can be regarded as biologically-adaptive responses that seek to promote self-relevant goals. However, sometimes they are either too intense or poorly matched to the demands of a current situation and thereby become dysfunctional. To control emotions does not imply to suppress any negative affect. Rather emotion regulation refers to a variety of automatic and controlled physiological, behavioral or cognitive processes through which individuals modulate both the experience and expression of emotions (Ochsner and Gross, 2005). The habitual use and the selection of particular regulation strategies differ among individuals. Moreover difficulties with emotion regulation have been suggested as a core mechanism underlying mood and anxiety disorders (Hermann et al., 2009). But, just as emotion regulation shapes a wide range of intra- and inter-personal processes, it can be itself subject to modification. As outlined above, the capacity to regulate emotions is particularly crucial when it comes to stressful situations, but the impact of acute stress and the associated neuroendocrine alterations on emotion regulation are less clear.

The stress response is characterized by the activation of two physiological systems: the fast-reacting sympathetic nervous system (SNS), resulting in the release of epinephrine and norepinephrine and the slower-reacting hypothalamic-pituitary-adrenal (HPA) axis which initiates a delayed release of glucocorticoids (GCs; de Kloet et al., 2005; Wolf, 2008; Joëls and Baram, 2009; Ulrich-Lai and Herman, 2009). Besides its effects at the periphery of the body, the main human GC cortisol influences multiple brain functions by activating GC-receptors located in the hippocampus, amygdala, and prefrontal cortical areas (de Kloet, 2003; McEwen, 2007; Lupien et al., 2009). For instance, it has been shown that stress enhances long term memory consolidation while impairing its retrieval (Roozendaal et al., 2006; Wolf, 2009).

Since the regions especially susceptible to stress (hippocampus, amygdala, and prefrontal cortex) are also known to be active during the effortful regulation of emotions (Ochsner and Gross, 2005) it appears reasonable to assume that acute stress may act as a modulating factor in the regulation of emotional responses. Of course, there is abundant evidence that initial release of stress hormones is typically associated with an increase in negative affect, while positive affective states are associated with a decrease in cortisol concentrations (Buchanan et al., 1999; Kuhlmann et al., 2005).

However, pharmacological studies in healthy participants have suggested that cortisol administered prior to a stressful event leads to attenuated negative affect ratings (Reuter, 2002; Het and Wolf, 2007). In line with this, patients with social phobia have been shown to report reduced anxiety in a psychosocial stress paradigm when treated with cortisone before stress exposure (Soravia et al., 2006). A recent integrative analysis using a laboratory stressor instead of pharmacological manipulations found an association between low negative affect and high cortisol concentrations in response to stress (Het et al., 2012). Taken together, these studies suggest a protective function of cortisol that might help to cope with the emotional load of a stressful situation (Het and Wolf, 2007). Less clear however is, which mechanism might be responsible for the buffering effects of cortisol on negative affect.

A first study directly relating stress and emotion regulation indicates that trait forms of suppression and reappraisal, two commonly used emotion regulation strategies (Ochsner and Gross, 2005), could be associated with elevated cortisol reactivity to a social-evaluative speech task (Lam et al., 2009). This is in line with some brain imaging studies, showing successful down-regulation of negative emotions to be mediated by the recruitment of prefrontal control areas inhibiting amygdala responses (Buhle et al., 2014).

Contrary to the proposed emotion-protective cortisol hypothesis however, it has been recently found that acute stress rather impaired newly acquired cognitive emotion regulation skills in a fear conditioning task (Raio et al., 2013). A possible explanation for the somewhat contradicting results might be the timing of the stressor, and with that different physiological stress systems being addressed to take an effect on emotion regulation.

In sum research still lacks studies explicitly focusing on the impact of acute stress on effortful emotion-regulatory processes. In the present study, we sought to explore how stress affects the application and effectiveness of three different emotion regulation strategies in a picture-based paradigm. Based on previous findings that address positive effects of cortisol on reducing negative affect in aversive situations (Het and Wolf, 2007; Het et al., 2012) we hypothesized that acute stress would facilitate the down-regulation of emotional responses to negative pictures. This would be in line with the idea, that increased cortisol reactivity to stressors might be linked to successful emotion regulation (Lam et al., 2009).

Evidence from studies investigating working memory suggests that stress often is associated with impaired working memory and less prefrontal mediated top down control (Arnsten, 2009). Moreover a recent neuroimaging study revealed that acute stress enhances the impairing impact of task-irrelevant emotional distractors (Oei et al., 2012). We thus hypothesized that stress impairs the effectiveness of distraction as an emotion regulation strategy.

Previous findings have demonstrated sex differences in the processing of emotional material (Bradley et al., 2001; Canli et al., 2002; Cahill and van Stegeren, 2003) and some studies observed sex-dependent influences of stress on cognition (Wolf et al., 2001; Schoofs et al., 2013). Moreover emotion research often revealed that women display stronger physiological and neuropsychological reactivity to emotional material than men (Lithari et al., 2010). Therefore, we additionally aimed to explore sex differences in the current emotion regulation task.

## Methods

### Participants

A total of 72 male and female students were recruited for participation in this study via advertisement and flyer at the Ruhr University Bochum. Participants were aged between 18 and 40 years (*M* = 24.8 years, *SD* = 5.1) and had a mean body mass index (BMI) of *M* = 22.2 kg/m^2^, *SD* = 2.5 kg/m^2^. Exclusion criteria checked beforehand in a telephone interview comprised use of hormonal contraceptives, smoking, chronic or acute illnesses, BMI outside the normal range between 18 and 26 kg/m^2^, and current medical or psychological treatment. In addition, we excluded students who had previously participated in the current stress protocol. All participants refrained from physical exercise and consumption of food and drinks except water at least 1 h prior to testing. The study was approved by the local ethics committee. All students provided written informed consent before participation and received a financial reimbursement of 15€ at the end of their testing session.

### Experimental design

A 2 × 2 × 3 design with the between-subject factors stress (stress vs. control), sex (women vs. men) and strategy (distract vs. increase vs. decrease) was used. We decided against the implementation of a within participant design in order to avoid carry over effects (e.g., if a participant first has to distance himself from a picture and afterwards should increase the emotional response to a picture). Participants were equally randomized to the stress- and the control group as well as to one of the three emotion regulation strategies by drawing lots. The resulting six experimental groups each comprised 12 participants (6 women and 6 men).

### Procedure and experimental paradigms

In order to control for circadian variations in cortisol concentrations experimental sessions were conducted between 1 and 6 p.m. First, participants signed a written informed consent form and filled out a demographic questionnaire. Subsequently participants were exposed either to a stressor or a control condition. After they rested for approximately 25 min, participants performed the emotion regulation paradigm (see below). In order to keep experimental conditions constant all participants were provided with simple crossword puzzles during the waiting period.

#### Stress and control procedure

In order to induce a stress response the socially evaluated cold pressor test (SECPT) was conducted as described in Schwabe et al. (2008). The stress protocol comprised immersion of the participant's right hand into a basin filled with ice-cold water (0–3°C) for 3 min while being videotaped and monitored by a reserved experimenter. In the control condition, participants immersed their right hand into a basin with warm water (36–37°C) without being videotaped or monitored.

#### Blood pressure, neuroendocrine, and subjective measurement

As a marker of SNS activity, blood pressure was measured 1 min before, during and 1 min after stress induction using Dinamap vital signs monitor (Critikon, Tampa, FL; cuff placed on the left upper arm).

We assessed free salivary cortisol concentrations at four different times; at baseline and 1, 25, and 45 min after completion of the SECPT or control condition. Saliva was collected using Salivette sampling devices (Sarstedt, Nümbrecht, Germany) and kept in a freezer until biochemical analysis. Free cortisol levels served as a measure of HPA axis activity and were determined by commercial immunoassays (CLIA; IBL International, Hamburg, Germany). Inter and intra assay variations were below 10%.

Directly after the stressor or control procedure participants rated on a scale from 0 (“not at all”) to 100 (“very much”) how stressful, painful and unpleasant they had felt during the procedure (rating method adopted from Schwabe et al., 2008).

#### Emotion regulation paradigm

A modified and combined version of previous designs to investigate emotion regulation was applied (Kanske et al., 2010). In this study, participants viewed negative and positive pictures (described below) and were asked to increase or decrease their upcoming emotional response by means of instructed techniques or required to solve an arithmetic task. The main focus of the increase condition was to intensify any emotional response elicited by the sample picture, for example, by imagining to be the person in that given situation, that it is happening at the moment or to think of all the consequences that might occur. In the decrease condition participants were instructed to distance themselves from the image by reinterpreting the entire situation, for example, to be only a photograph or produced by actors and therefore not real. Similar to previous research (Kanske et al., 2010; McRae et al., 2010) the distract condition provided participants with an arithmetic task, presented as a transparent overlay and required them to decide whether the displayed solution was correct or not. All arithmetic problems were formed with 2 operands including a subtraction or an addition and were randomly assigned to the picture background condition (positive vs. negative). After each picture presentation, participants rated their current emotional reactions on a 9-point scale using the Self-Assessment-Manikin (SAM) scales for subjectively experienced arousal and valence. The SAM is a non-verbal self-assessment technique developed by Bradley and Lang (1994), which is commonly used in emotion research (Rösch et al., 2013; Adam et al., 2014). Written instructions for the application of the three different emotion regulation strategies as well as for the emotional rating were provided beforehand. Each trial started with the presentation of a picture (10 s), which served both as the emotion induction and the emotion regulation phase. Subsequently the three SAM-scales emerged on the computer screen. The completion of the emotional rating was directly followed by the next picture presentation, except in the distract condition. There participants first rated their emotional response then indicated whether the equation was correct or incorrect and subsequently the next picture emerged. Neither participant's answers on the emotional rating nor on the correctness of the arithmetic problem were limited by time. Feedback on the response accuracy was not provided. In total, the paradigm consists of 60 trials (30 negative and 30 positive stimuli) and lasted about 30 min.

#### Stimuli

Pictures were selected from the International Affective Picture System (IAPS) based on normative ratings in valence and arousal (Lang et al., 2008). Sets of 30 negative and 30 positive pictures were created. Negative and positive stimuli were both highly arousing but differed in regard to valence, as indicated by *t*-tests [arousal: *t*_(58)_ = 0.57, *p* = 0.57and valence: *t*_(58)_ = 20.62, *p* < 0.001, respectively]. Differences in luminance and complexity were kept minimal. The presentation order of the two picture blocks was counterbalanced over participants.

### Statistical analysis

For all statistical tests, the level of significance was set to 0.05. Blood pressure, cortisol and behavioral data were analyzed using repeated measurement analyses of variance (ANOVAs) as described in detail in the respective result sections. Greenhouse-Geisser corrected *p*-values were reported if the assumption of sphericity was violated. *Post hoc* tests were performed using exploratory *t*-tests. *P*-values were corrected for unequal variances if appropriate.

## Results

### Stress response

#### Salivary cortisol

Compared to the control condition, the stress group showed a significant increase in salivary cortisol concentrations in response to the SECPT, as indicated by a significant time × stress interaction [*F*_(2, 115)_ = 4.55, *p* = 0.016] in a 4 × 2 × 2 ANOVA with the within-subject factor time (baseline, +1 min, +25 min, +45 min) and the between-subject factors stress and sex. In addition a significant main effect of time [*F*_(2, 115)_ = 7.75, *p* = 0.001], as well as a significant main effect of stress [*F*_(1, 65)_ = 4.68, *p* = 0.034] emerged. As illustrated in Table 1, *t*-tests revealed that the stress group (compared to the control group) displayed significantly higher cortisol concentrations 25 and 45 min after the SECPT/control condition [*t*_(50)_ = 2.77, *p* < 0.01 and *t*_(70)_ = 2.36, *p* = 0.02, respectively], while the groups did neither differ at baseline (*p* = 0.43) nor 1 min after the treatment (*p* = 0.35).

**Table 1:** **Salivary cortisol concentrations and blood pressure responses to as well as subjective ratings of the SECPT vs. control procedure**.

	**Stress**	**Control**
**SALIVARY CORTISOL (nmol/l)**		
Before treatment	9.55 ± 1.18	8.65 ± 0.74
1 min after treatment	10.63 ± 1.31	9.08 ± 0.98
25 min after treatment	12.51 ± 1.53^**^	7.71 ± 0.80
45 min after treatment	8.90 ± 0.85^*^	6.46 ± 0.59
**SYSTOLIC BLOOD PRESSURE (mmHg)**		
1 min before treatment	127.00 ± 2.81	124.70 ± 2.97
During treatment	144.20 ± 3.29^***^	121.70 ± 2.57
1 min after treatment	121.50 ± 3.01	117.30 ± 2.49
**DIASTOLIC BLOOD PRESSURE (mmHg)**		
1 min before treatment	70.30 ± 1.12	69.90 ± 1.44
During treatment	88.70 ± 1.65^***^	72.00 ± 1.40
1 min after treatment	68.80 ± 1.37	66.90 ± 1.37
**SUBJECTIVE RATINGS AFTER TREATMENT**		
Unpleasantness	58.61 ± 4.05^***^	6.39 ± 2.62
Stressfulness	46.94 ± 4.40^***^	3.33 ± 1.20
Painfulness	63.61 ± 4.07^***^	2.50 ± 1.22

*Data represents means ± s.e.m*.

*Significant difference between stress and control group*,

* p < 0.05, and

** *p < 0.01*,

*** *p < 0.001 (t-tests)*.

**Figure 1:**
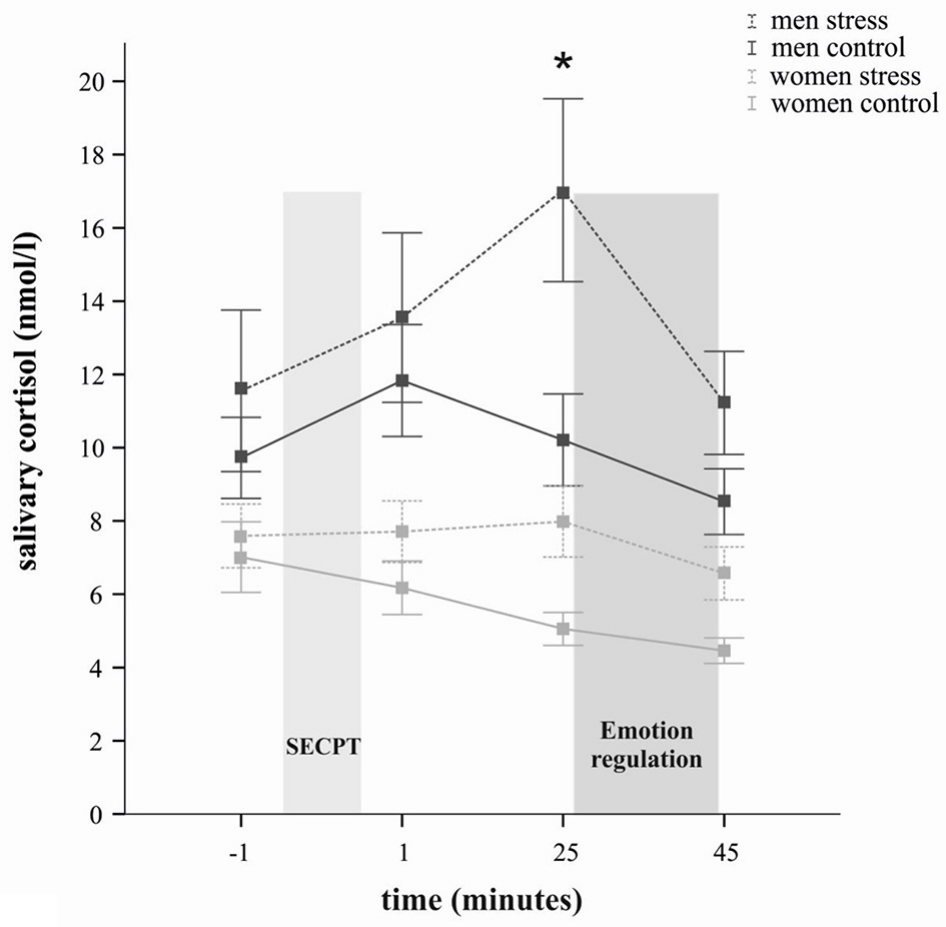
**Salivary cortisol in nanomoles per liter (*M* ± s.e.m.) at several time points across the experiment**. The lighter gray bar represents the time of the stress and control manipulation, respectively; the darker gray bar represents the time of the emotion regulation paradigm. Cortisol concentrations were significantly increased in the socially evaluated cold-pressor test (SECPT) groups but not in participants exposed to the warm water control condition. Men showed overall higher cortisol concentrations than women. In comparison to men from the control group, stressed men displayed significantly higher cortisol concentrations 25 min after the stress manipulation (^*^p < 0.05; *t*-test). Similarly in comparison to women in the control group, stressed women displayed significantly higher cortisol concentrations 25 min after stress manipulation (^*^p < 0.05; *t*-test).

Further, cortisol responses were determined not only by stress induction itself, but showed sex-dependent differences, as reflected by a significant time × sex interaction [*F*_(2, 115)_ = 4.55, *p* = 0.016] and a significant main effect of sex [*F*_(1, 65)_ = 19.34, *p* < 0.001]. The time × stress × sex interaction did not reach significance (*p* = 0.31). Overall, women compared to men displayed significantly lower cortisol concentrations at all measurements, as indicated by follow-up *t*-tests (all *p*s < 0.05). In regard to the stress group only, exploratory *t*-tests revealed, that whereas at baseline (*p* = 0.17) the cortisol levels did not differ between men and women, the stress response in men (compared to women) was accompanied by significantly larger cortisol concentrations at all three post-treatment time points (all *p*s < 0.05) (see Figure 1). As Figure 1 illustrates, stressed women did not show a robust cortisol increase but rather stable cortisol concentrations. They displayed, however, significantly higher cortisol levels than women in the control group 25 min after stress manipulation [*t*_(32)_ = 2.75, *p* = 0.01].

Since cortisol concentrations are lower in the evening we additionally controlled for time of day variable in order to examine whether women were tested disproportionally later in the day. There was neither a sex nor a stress main effect [*F*_(1, 68)_ = 0.013, *p* = 0.911 and *F*_(1, 68)_ = 0.28, *p* = 0.60, respectively]. Moreover the two factors did not interact (*p* > 0.05). In addition bivariate correlations between the time of day variable and the cortisol increase delta variable (25 min—baseline) showed that time of day was not correlated with cortisol responses to the SECPT (*p* = 0.53). Thus, within the time frame of this study time of day appears not to have had a strong influence on HPA activity.

#### Blood pressure

The SECPT elicited a significant increase in both systolic and diastolic blood pressure in stressed participants compared to controls (see Table 1). A 3 × 2 × 2 ANOVA with the factors time, stress and sex revealed a significant time × stress interaction [systole: *F*_(2, 136)_ = 54.20, *p* < 0.001 and diastole: *F*_(2, 111)_ = 55.64, *p* < 0.001]. In addition significant main effects of time [systole: *F*_(2, 136)_ = 79.85, *p* < 0.001 and diastole: *F*_(2, 111)_ = 121.03, *p* < 0.001], stress [systole: *F*_(1, 68)_ = 10.24, *p* = 0.002 and diastole: *F*_(1, 68)_ = 14.91, *p* < 0.001] and sex [systole: *F*_(1, 68)_ = 45.02, *p* < 0.001 and diastole: *F*_(1, 68)_ = 7.19, *p* = 0.009] emerged. *T*-tests indicated significantly higher systolic and diastolic blood pressure in the stress compared to the control group during hand immersion only [*t*_(70)_ = 5.40, *p* < 0.001 and *t*_(70)_ = 7.73, *p* < 0.001, respectively]. Furthermore, blood pressure in men was constantly higher than in women at all measurement points, as indicated by follow-up *t*-tests (all *p*s < 0.022). However, there was no significant interaction with the factor sex.

#### Subjective ratings

Compared to the control procedure, participants of the stress group experienced the SECPT as significantly more stressful, painful and unpleasant, as indicated by significant main effects of stress in 2 × 2 ANOVAs (all *F*s > 90.64, all *p*s < 0.001) (see Table 1). No other main or interactions effects were significant. Men and women did not differ with respect to their subjective stress ratings (all *F*s < 0.45 *p*s > 0.24).

### Stress effects on emotion regulation

A table containing all the descriptive results can be found in the supplement. To assess the performance on emotion regulation we conducted a 2 × 2 × 3 × 2 ANOVA with picture valence (negative vs. positive) as within-subject factor and three between-subject factors separately for arousal and valence ratings; stress (stress vs. control group), strategy (distract vs. increase vs. decrease) and sex (men vs. women).

For arousal the analysis revealed a main effect of strategy [*F*_(2, 60)_ = 10.39, *p* < 0.001]. As to be expected the increase and decrease conditions significantly differed from each other. The distract condition was associated with intermediate ratings which only tended to differ from the other two conditions. In addition there was a picture valence by sex interaction [*F*_(1, 60)_ = 9.26, *p* < 0.01] with women reporting more arousal to negative pictures and less arousal to positive pictures when compared to men. Furthermore, there was a trend toward a four-way interaction between picture valence, stress, strategy and sex [*F*_(2, 60)_ = 2.47, *p* = 0.09].

**Figure 2:**
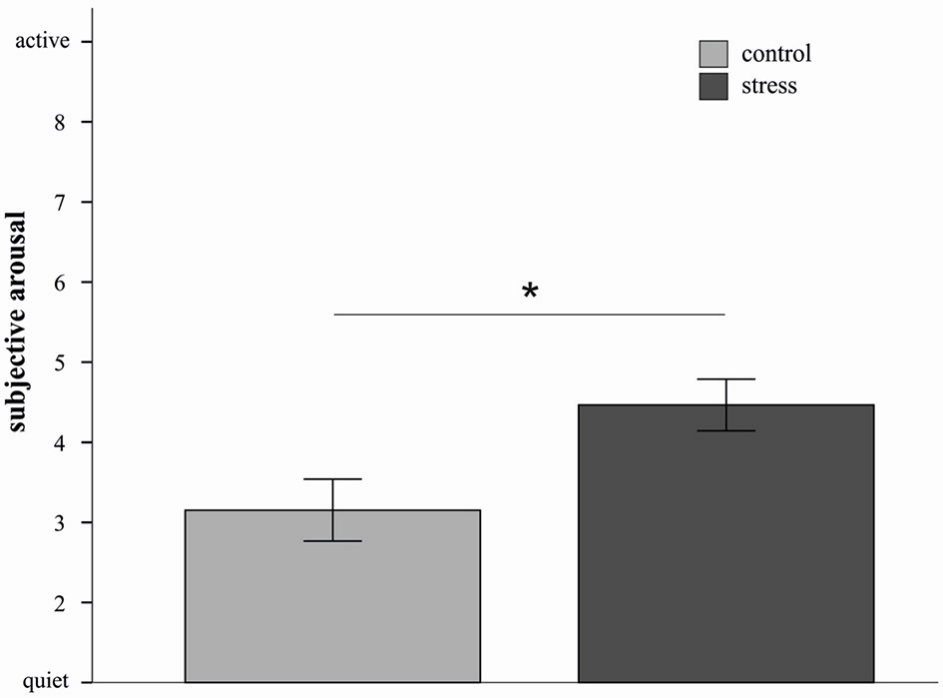
**Mean emotional ratings (±s.e.m.) to sample pictures in the distract condition are depicted for the stress and control group**. Stressed participants in the distract condition reported higher subjective arousal compared to controls. ^*^p < 0.05 (*F*-test).

In order to characterize the four-way interaction further and based on the consideration that the impact of stress might differ depending on the used strategies, 2 × 2 × 2 ANOVAs with the factors picture valence, stress and sex were conducted for the three emotion regulation strategies separately. Results indicated a significant main effect of stress for the distract condition [*F*_(1, 20)_ = 7.13, *p* = 0.02, *d* = 1.11]. As depicted in Figure 2, stressed participants reported higher subjective arousal ratings compared to non-stressed participants. In addition a sex by valence interaction was again observed [*F*_(1, 20)_ = 9.10, *p* < 0.01]. The picture valence × stress × sex interaction did not reach significance (*p* = 0.15).

The ANOVAs for the other two emotion regulation condition did not reveal significant effects for the factors stress or sex.

For the subjective valence ratings a similar four factorial ANOVA was conducted. Results showed a significant picture valence by sex interaction [*F*_(2, 60)_ = 5.85, *p* = 0.02] with women reporting lower valence ratings for negative pictures as well as higher valence ratings for positive pictures compared to men. Furthermore, a significant picture valence by strategy interaction [*F*_(2, 60)_ = 14.46, *p* < 0.001] and a significant sex by strategy interaction [*F*_(2, 60)_ = 3.18, *p* = 0.05] was observed. Subjective valence ratings in the increase condition were significantly different from the decrease and distract condition with lower reported valence for negative pictures and higher valence for positive pictures. With respect to stress a significant stress × strategy × sex interaction [*F*_(2, 60)_ = 4.0, *p* = 0.02] was found.

**Figure 3:**
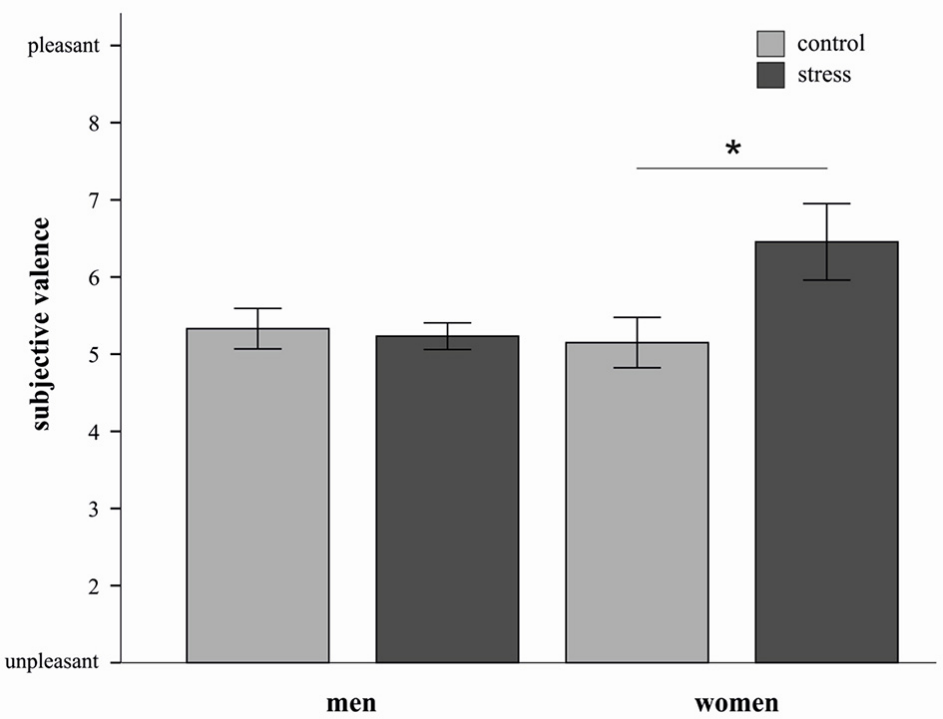
**Mean emotional ratings (±s.e.m.) to sample pictures in the decrease condition are depicted for both sexes in the stress and control group**. Stressed women in the decrease condition reported higher subjective valence than control women ^*^p = 0.05 (*t*-test).

In order to characterize this three way interaction further we conducted three separate ANOVAs (for each strategy) with the factors stress and sex. For the decrease condition a significant stress by sex interaction was observed *F*_(1, 20)_ = 4.36, *p* = 0.05). Stressed women reported higher subjective valence ratings compared to women from the control group [*t*_(10)_ = −2.2, *p* = 0.05, *d* = 1.28] (see Figure 3). In men no such effect was apparent.

No significant stress effects on valence ratings were observed in the other two strategy conditions.

### ANCOVAS

In order to characterize the impact of the stress induced cortisol increase and in order to pay tribute to the differences in stress induced cortisol concentrations between the sexes we conducted ANCOVAS with the cortisol increase delta variable (25 min—baseline) as covariate.

For subjective arousal the four way ANCOVA revealed a trend toward a stress by strategy interaction (*p* = 0.07) and a significant four way interaction [stress by sex by strategy by valence (*p* < 0.05). The three way ANCOVA for the distraction condition again showed a main effect of stress *F*_(1, 18)_ = 5.24, *p* < 0.05].

For subjective valence the four way ANCOVA revealed a significant main effect of treatment [*F*_(1, 20)_ = 4.16, *p* < 0.05]. This main effect was further specified by a significant three way interaction of the factors stress, sex, and strategy [*F*_(1, 20)_ = 5.27, *p* < 0.01]. The two way ANCOVA (stress and sex) for average subjective valence within the decrease condition again revealed a sex by stress interaction [*F*_(1, 18)_ = 5.01, *p* < 0.05].

In sum the ANCOVAs supported the conclusions drawn from the ANOVAs and highlighted the fact that the impact of stress observed in the subjective ratings was not secondary to the stress induced cortisol increases.

## Discussion

The aim of the present study was to investigate the effects of acute stress on the effectiveness of three different strategies to regulate emotional responses toward negative and positive pictures. As confirmed by blood pressure measures, subjective ratings and salivary cortisol data, stress induction prior to emotion regulation was successful. However, it has to be noted that cortisol only increased substantially in stressed men but not in stressed women. We found that stress impaired the effectiveness of distraction, as indicated by higher self-reported arousal of stressed participants. This effect was not further modulated by sex or picture valence even though these factors interacted with each other.

Similar to our results, a recent fMRI-study revealed that after acute social stress increased attention to emotional distractors slowed down working memory performance (Oei et al., 2012). Moreover, in stressed participants emotional distraction was associated with a stronger activation in ventral affective areas than in controls. Consistent with these findings, Roelofs and colleagues found increased selective attention for angry faces in an emotional stroop task in high cortisol responders relative to low cortisol responders and non-stressed participants (Roelofs et al., 2007). In anxious individuals this stress-induced hyper vigilance for emotional threat cues seems to be especially prominent (van Peer et al., 2007).

In addition our results are well in line with findings that imply cognitive flexibility (Plessow et al., 2012) to be impaired under stress. For instance, a recent study revealed a correlation between stress-induced cortisol increases and pronounced between-task interference in a dual-task paradigm (Plessow et al., 2012). Results further suggest that the observed inability to protect processing of a prioritized task over manipulations from a second task might be caused by reduced task shielding in stressed participants. Moreover, our findings are consistent with work in humans and monkey showing that social stressors lead to impaired PFC functioning and rapid alterations in both executive functioning and working memory performance (Arnsten, 2009). The stress-induced impairments found in the current study therefore suggest that distraction as a cognitive emotion regulation strategy may be largely ineffective in the face of stress.

Still, our findings contrast previous pharmacological studies indicating that an administration of cortisol reduces the susceptibility to goal-irrelevant emotional distractors in a working memory task (Putman and Roelofs, 2011). However, when comparing the present study with pharmacological cortisol studies it is important to emphasize that our stressor activated both the SNS and the HPA axis (Schwabe et al., 2008). Inconsistencies between stress and cortisol studies therefore may rather emphasize that acute stress and a sole administration of cortisol does not necessarily lead to the same outcome.

We found that stress enhanced the ability to decrease negative emotions in women. These results are in line with previous findings that suggest cortisol to reduce negative affect in response to aversive situations in the aftermath of stress (Reuter, 2002; Het and Wolf, 2007; Het et al., 2012). Our findings also mirror data from clinical studies demonstrating that the administration of cortisol can effectively attenuate anxiety toward threat-related stimuli in both spider- and social-phobic patients (Soravia et al., 2006).

However, since the findings for the decrease condition did only occur in stressed women who did not show a robust cortisol response to the SECPT other mediating pathways need to be considered. During the SECPT participants have to overcome their urge to remove the hand from the ice cold water and thus have to tolerate the occurring pain. This experience might help to deal with negative stimuli in the aftermath of stress.

Research on social cognition further support the idea that women respond with affiliative behavior when confronted with moderately stressful circumstances and that this effect may be oxytocin mediated (Taylor, 2006). Oxytocin is associated with parasympathetic functioning, suggesting a counterregulatory role in psychological stress responses (Taylor et al., 2000). Since its release in response to stress appears to be larger in women than in men (Taylor et al., 2000) oxytocin might be considered as a potential mechanism mediating the currently observed sex-specific beneficial effects of acute stress on down-regulating negative emotions.

The observed sex differences in response to the SECPT warrant an additional discussion. We found women to display significantly lower cortisol levels in response to stress than men. Previously it has been shown that in the Trier Social Stress Test men and naturally cycling women only respond with cortisol increases when confronted with an evaluative committee that was composed of the opposite sex (Duchesnea et al., 2012). Since the SECPT in the present study was conducted by a female experimenter, it could be suggested that this contextual factor of the stress manipulation might have dampened individual cortisol responses in women. Future SECPT studies should consider controlling for experimenter's sex more systematically.

For the current results it is, however, important to note that the sex differences in emotion regulation still remained significant after inclusion of delta cortisol as a covariate and therefore couldn't be solely explained by the different cortisol stress reactivity in men and women. Furthermore, all other stress markers did not show any sex differences. Together with previous findings our results provide initial evidence that the impact of stress on cognitive emotion regulation may be different for men and women at least with respect to the ability to decrease emotions intentionally.

Our finding of a stress- induced increase in emotion regulation capacities in women is in contrast to a recent study using a fear conditioning paradigm to investigate the effects of acute stress on the effectiveness of a previous learned emotion regulation technique (Raio et al., 2013). In contrast to controls, stressed participant were impaired to reduce fear responses toward the conditioned aversive cue despite precedent emotion regulation training. Moreover, regulated fear responses were correlated with salivary α-amylase 10 min after the stressor. These findings suggest that early catecholamine responses driven by SNS arousal may be one mechanism mediating the detrimental effects of acute stress on emotional down-regulation.

It is worth emphasizing, that the detrimental effects of acute stress on distraction were apparent in the arousal but not in the valence ratings, whereas it was the other way round for the decrease condition, with significant results emerging only in the valence dimension. These findings suggest that acute stress might influence different emotion regulation processes specifically for valence and arousal. This would fit into a theoretical framework, proposed by the circumplex model of affect (Posner et al., 2005), which assumes that valence and arousal underlie two independent neurophysiological systems, generating a complex emotional response.

The present study has several limitations which need to be acknowledged. We had decided to design the factor emotion regulation strategy as a between group factor. The rationale behind this decision was the aim to avoid potential carry over effects in the case of repeatedly changing emotion regulation strategies (as would have been the case in a within subject condition) (Lamke et al., 2014). Due to these restrictions a blocked design has been already used in previous studies investigating emotion regulation processes (Banks et al., 2007; Bebko et al., 2011; Kim and Hamann, 2012). However, the downside of this approach was the rather small number of participants in each condition. Our study was with an *n* of 72 participants rather large for a neuroendocrine stress study, however each cell defined by the factors stress, sex, and strategy only contained six participants. Especially the obtained sex by stress interaction for the decrease strategy has therefore to be considered preliminary.

In order to obtain a reliable measure of subjective emotional reactivity we used a total of 60 pictures (30 negative and 30 positive ones). This scenario is of course rather artificial even though it is not uncommon in this area of research (Kanske et al., 2010, 2012; Kim and Hamann, 2012; Ahn et al., in press).

We relied on self-reported measures of arousal, in line with previous studies on the topic of emotion regulation. It would have been informative to combine these self-reported measures with physiological markers (e.g., skin conductance, heart rate variability, pupil dilation) (Urry et al., 2009; Bebko et al., 2011; Kim and Hamann, 2012; Raio et al., 2013; Vanderhasselt et al., 2014).

A further limitation to this study is that we did not assess data concerning sex hormones or menstrual cycle phase in female participants. Since previous research suggest that sex and hormonal contraceptive use have effects on HPA and autonomic nervous system responsiveness to acute psychosocial stress (Kirschbaum et al., 1995; Kudielka and Kirschbaum, 2005; Kajantie and Phillips, 2006) as well as on emotional memory processes (Derntl et al., 2008; Guapo et al., 2009; Milad et al., 2010; Merz et al., 2012; ter Horst et al., 2012) future studies should take these issues into account. Further investigation into whether these factors also modulate stress-induced emotion regulation impairments or improvements, respectively, will be critical to fully characterize how stress may affect emotion regulation processes differently in men and women.

Despite these limitations, this is the first study directly linking acute stress with the differential success of particular emotion regulation strategies. We provide evidence that the use of distraction appears to be impaired by stress. Moreover we provide initial evidence for the hypotheses that stress may support some emotion regulatory processes (decrease), which in turn may protect the individual from emotional disturbances after stressful situations (Het and Wolf, 2007; Het et al., 2012). These findings imply that there might be emotion regulation strategies which improve in the aftermath of acute stress while other strategies may rather be impaired under stressful conditions. In the long run future research on that topic may contribute to a distinct characterization of emotion regulation strategies that are especially effective in the post stress phase.

### Conflict of interest statement

The authors declare that the research was conducted in the absence of any commercial or financial relationships that could be construed as a potential conflict of interest.
