# Supplementary material for: Emotion regulation: exploring the impact of stress and sex
Source: Front Behav Neurosci. 2014 Nov 13;8:397. doi: 10.3389/fnbeh.2014.00397 (PMC4230035; doi:10.3389/fnbeh.2014.00397)
Supplement: Supplementary file 1 [file Table1.DOCX]

**Supplementary Material**

**Emotion regulation: Exploring the impact of stress and sex**

**Supplementary Table 1.** *Self-reported arousal and valence ratings (M ± SEM) to negative and positive pictures are depicted for both sexes in the stress and control groups. Data is shown separately for each emotion regulation strategy condition (distract, increase and decrease).*

|  |  |  | **control** | |  | **stress** | |
| --- | --- | --- | --- | --- | --- | --- | --- |
|  |  |  | men | women |  | men | women |
| **Arousal** | negative | distract | 3.10 ± 0.72 | 4.79 ± 0.67 |  | 4.40 ± 0.61 | 5.86 ± 0.19 |
|  |  | increase | 5.69 ± 0.66 | 4.78 ± 0.73 |  | 4.57 ± 0.69 | 6.19 ± 0.13 |
|  |  | decrease | 3.96 ± 0.92 | 3.04 ± 0.77 |  | 3.30 ± 0.24 | 3.58 ± 0.58 |
|  | positive | distract | 2.64 ± 0.65 | 2.08 ± 0.33 |  | 3.46 ± 0.58 | 4.14 ± 0.43 |
|  |  | increase | 4.77 ± 0.64 | 4,02 ± 0.85 |  | 3.77 ±0.85 | 3.99 ± 0.62 |
|  |  | decrease | 2.62 ± 0.64 | 1.65± 0.21 |  | 3.17 ± 0.33 | 2.31 ± 0.55 |
| **Valence** | negative | distract | 4.02 ± 0.33 | 3.01 ± 0.36 |  | 3.77 ± 0.15 | 2.84 ± 0.27 |
|  |  | increase | 2.68 ± 0.21 | 2.87 ± 0.26 |  | 3.51 ± 0.26 | 2.76 ± 0.11 |
|  |  | decrease | 4.14 ± 0.53 | 3.65 ± 0.20 |  | 4.10 ± 0.15 | 5.04 ± 0.68 |
|  | positive | distract | 7.34 ± 0.51 | 7.86 ± 0.31 |  | 7.52 ± 0.24 | 7.58 ± 0.34 |
|  |  | increase | 7.51 ± 0.21 | 7.61 ± 0.43 |  | 7.46 ± 0.27 | 7.71 ± 0.25 |
|  |  | decrease | 6.52 ± 0.37 | 6.65 ± 0.76 |  | 6.37± 0.36 | 7.87 ± 0.45 |
